# Cryptococcal Meningitis in an Immunocompetent Man Exposed to a Pet Cockatoo: An Overlooked Zoonosis

**DOI:** 10.7759/cureus.28122

**Published:** 2022-08-17

**Authors:** Diego P Peralta, Haya Najjar, Jessica Garcia-Chan

**Affiliations:** 1 Infectious Diseases, Texas Tech University Health Sciences Center El Paso, El Paso, USA; 2 Department of Medicine, University of California San Diego, San Diego, USA; 3 Neurology, Texas Tech University Health Sciences Center El Paso, El Paso, USA

**Keywords:** pet cockatoo, immunocompetent, cryptococcus neoformans var. grubii, bird, zoonotic transmission, meningitis

## Abstract

*Cryptococcus *species are commonly isolated in the excreta of birds, but zoonotic transmission has yet to be proven. We report a case of an immunocompetent man with meningitis caused by *Cryptococcus neoformans *var. *grubii *with significant exposure to a pet cockatoo highly suspicious for zoonotic transmission. Treatment with intravenous liposomal amphotericin B and oral flucytosine was initiated upon diagnosis, but diagnostic delay because of low suspicion contributed to neurological sequelae. Recognition of pet birds as potential sources of *Cryptococcus* species' zoonotic transmission is essential for prompt diagnosis and treatment.

## Introduction

*Cryptococcus* species are isolated from bird droppings [1,2], but the potential for zoonotic transmission remains uncertain. Few cases investigate pet birds as a possible source of zoonotic transmission of *C. neoformans*, most being observed in people with underlying immunodeficiency or malignancy [3-5]. We report a case of *C. neoformans* var. *grubii* meningitis in an immunocompetent person with significant exposure to a pet cockatoo supporting zoonotic transmission.

This article was previously presented as an abstract and poster at the 2022 American Society of Microbiology Rio Grande Branch Meeting on April 14, 2022.

## Case presentation

A 78-year-old man presented with a one-year history of gait instability that progressed to recurrent falls three weeks before hospitalization. There were no reports of fever, headaches, neck pain, visual changes, dizziness, or modification of his mental status. His medical history included hypertension, deep vein thrombosis, pulmonary embolism, depression, and latent tuberculosis infection (LTBI), for which he was on his eighth month of treatment with isoniazid and pyridoxine. He had a workup initiated by an outpatient neurologist one year ago. MRI brain showed leptomeningeal enhancement. Cerebrospinal fluid (CSF) analysis showed elevated protein and lactate dehydrogenase (LDH), low glucose (Table 1), non-reactive venereal disease research laboratory (VDRL), and negative *Mycobacterium tuberculosis* complex polymerase chain reaction (PCR) and cultures. CSF cell count and differential were not performed. QuantiFERON®-TB Gold Plus (QIAGEN, Hilden, Germany) was positive. No CSF or serum testing for *Cryptococcus* or other pathogens was performed. He was referred to the local Department of Public Health for LTBI treatment. Repeated MRI brain after three months showed persistent leptomeningeal enhancement, but no additional workup was obtained. His symptoms worsened over the year, limiting his ability to independently complete activities of daily living.

**Table 1:** Cerebrospinal fluid analysis H: High, L: Low

	Reference Range	Outpatient Lumbar Puncture	Inpatient Lumbar Puncture #1	Inpatient Lumbar Puncture #2
Opening pressure	10-20 cm H2O	N/A	N/A	13
Appearance spun		N/A	Pale yellow	Colorless
Appearance		N/A	Hazy	Clear
Red blood cells	<5 /µL	N/A	10 (H)	0
White blood cells	<5 /µL	N/A	285 (H)	112 (H)
Neutrophils	0%	N/A	23 (H)	8 (H)
Lymphocytes	0%	N/A	68 (H)	61 (H)
Macrophages	3-37%	N/A	6	26
Plasma cells	%	N/A	3	5
Glucose	40-70 mg/dL	38 (L)	36 (L)	46
Protein	12-60 mg/dL	464 (H)	284 (H)	198 (H)
Lactate dehydrogenase	<=25 U/L	44 (H)	55 (H)	26 (H)
Cryptococcal antigen	Negative	N/A	1:320 (H)	1:5 (H)

**Figure 1:**
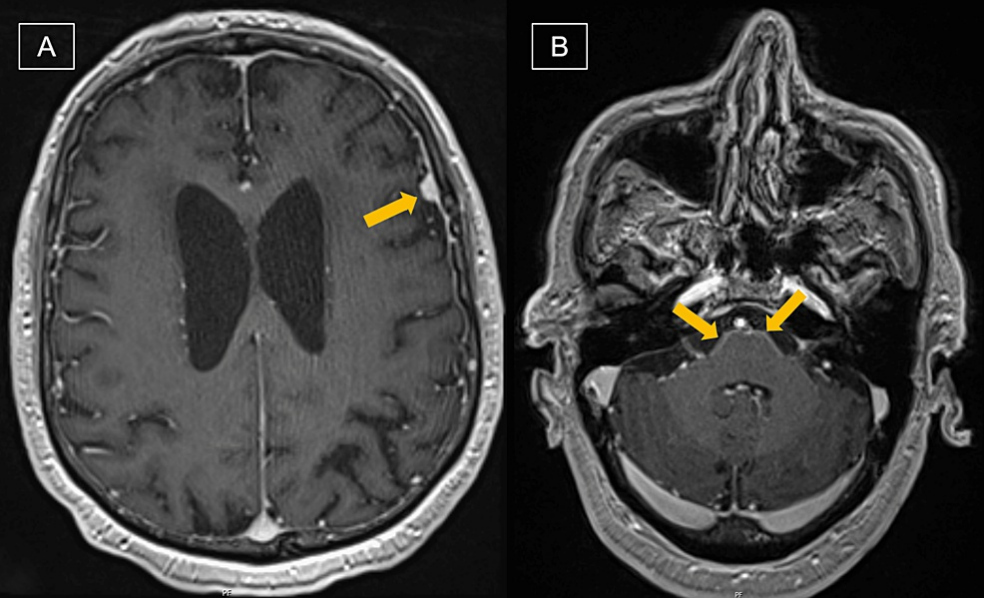
MRI brain with contrast (A) Bifrontal/bitemporal pachymeningeal enhancement with nodularity with a broad-based 7 mm nodule (arrow) and (B) leptomeningeal enhancement prepontine cistern (arrows)

**Figure 2:**
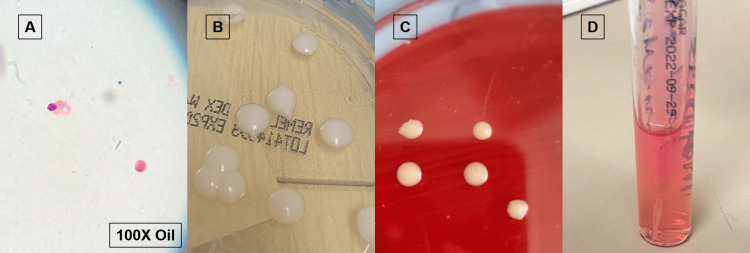
Cryptococcus neoformans var. grubii (A) Cerebrospinal fluid (CSF) gram stain at 100x magnification, (B) Colonies in Sabouraud Dextrose agar, (C) Brain heart infusion agar, and (D) Urease tests positive

Examination revealed an overweight man in mild distress with a temperature of 36.9°C, a pulse of 86 beats/minute, a blood pressure of 192/83 mmHg, and a respiratory rate of 17 breaths/minute. He had diminished strength in the lower extremities with significant spasticity, full strength in the upper extremities, and an intention tremor of the right upper extremity. No meningeal signs were found. Cranial nerves, reflexes, sensation, and orientation were intact. MRI brain with contrast revealed bifrontal/bitemporal predominant smooth pachymeningeal enhancement with nodularity along the left frontal convexity dura with a broad-based 7 mm nodule and leptomeningeal enhancement along the basal cisterns, predominantly prepontine and premedullary cisterns (Figure 1). HIV screening was negative. CSF analysis showed elevated white blood cell count with lymphocyte predominance, elevated protein and LDH, and low glucose (Table 1). CSF cryptococcal antigen (CrAg) was positive. *Cryptococcus* was also detected in BioFire (BioFire Diagnostics, Utah, United States). Within 72 hours, a yeast grew in both Sabouraud Dextrose and brain heart infusion agar, later identified as *C. neoformans* var. *grubii* by matrix-assisted laser desorption/ionization-time of flight (MALDI-TOF) mass spectrometry (MS) with a log (score) value of 2.25. CSF urease test was positive (Figure 2). Serum CrAg and fungal blood culture were negative. 

Infectious diseases (ID) consultation was requested upon diagnosis. During the ID evaluation, the patient denied a prior diagnosis of immunodeficiency, opportunistic infection, past hospitalizations due to an infectious process, or the use of immunosuppressive drugs. He also denied a family history of immunodeficiencies or a recent travel to a tropical or subtropical region. Further questioning revealed that he cared for a pet cockatoo caged indoors for about 10 years. He spent most of the day with the bird, kissed her, fed her, cleaned after her, and on multiple occasions, the bird defecated on him. The cockatoo developed an unknown illness characterized by bloody excreta and feather loss passing away a few months before the patient’s onset of symptoms. Otherwise, he had no exposure to other birds, soil, job hazards, or sick contacts. Given HIV seronegative status, immunodeficiency and malignancy workup were obtained but did not yield significant findings.

The patient was started on induction liposomal amphotericin B and flucytosine. Induction therapy was complicated by acute kidney injury, managed with hydration and correction of electrolyte derangements. In the third week of treatment, a repeated lumbar puncture showed normal opening pressure, reduced white blood cell count, persistent lymphocyte predominance, reduced protein level, normal glucose level, and decreased LDH. CSF CrAg was positive with a lower titer (Table 1). CSF culture was negative. During this time, he had improvement in lower extremity spasticity and right upper extremity tremor, but gait instability persisted. After four weeks, he was transitioned to consolidation therapy with high-dose fluconazole (800 mg daily) with a tentative duration of eight weeks. Maintenance therapy with low-dose fluconazole (200 mg daily) is anticipated for six to 12 months.

## Discussion

Zoonotic transmission of *Cryptococcus* species has been suspected, given a strong association of *Cryptococcus* with birds, but limited evidence exists. Furthermore, the risk associated with exposure to pet birds is unclear. Few preceding case reports demonstrate associations between pet birds and cryptococcal infections of those exposed [3-6]. Only one case report, in 2005, reported such findings in an immunocompetent patient and was supported by isolation of the same *Cryptococcus* species in the pet bird excreta by molecular testing as that causing infection in the patient [6]. A case report in 2000 also isolated the same species in pet bird excreta by molecular testing as that infecting an immunocompromised patient [3]. In both case reports, neither the immunocompetent patient nor the immunocompromised patient was in physical contact with the pet bird or its excreta [3,6]. In contrast, our patient had direct repeated daily exposure to the excreta of his pet cockatoo and potential aerosolized particles in the presence of the indoor birdcage [7,8]. Furthermore, he was exposed for 10 years in comparison to seven years and three months in the 2000 and 2005 cases reports, respectively [3,6]. This suggests that the patient had prolonged exposure to potentially acquire the infection from his pet bird in the absence of other exposures.

Cryptococcal meningitis was not considered during the initial workup, given the patient’s immunocompetent status despite a year-long history of neurological symptoms. HIV seronegative status further reduced suspicion of cryptococcal infection. Immunocompetent people with cryptococcal meningitis have a delay in diagnosis due to low suspicion of cryptococcal infection [9-12]. Such delay may be attributed to premature closure and failure to recognize atypical presentations of cryptococcal meningitis [9,12]. It was only when CSF CrAg was positive, followed by fungal growth, that the diagnosis was obtained, highlighting the importance of CrAg testing in those presenting with lymphocytic meningitis. If travel history is reported, tropical infections should also be considered. Keeping a broad differential plays a critical role in obtaining an earlier diagnosis. Consequently, the patient was not started on appropriate treatment until over a year after the onset of symptoms. 

In addition to his immunocompetent status, his nonspecific presentation may have also contributed to a delay in diagnosis. Compared to immunodeficient people, immunocompetent people who acquire cryptococcal meningitis are less likely to be febrile or have a headache [11-13]. Furthermore, people with known immunodeficiencies are more likely to have an earlier evaluation by ID consultants than immunocompetent people [12]. Delay in ID evaluation has been associated with increased mortality in people with cryptococcal infection [12,14]. 

When comparing neurological outcomes between immunocompromised and immunocompetent groups with cryptococcal meningitis, the latter group showed higher mortality and more significant neurological sequelae [10-13]. This is thought to be related to diagnostic delay and paradoxical worsening attributed to an intact immune system [15]. Although the patient improved based on repeated CSF analysis and reduction in lower extremity spasticity, he remained at high risk of falls due to persistent gait instability.

## Conclusions

Cryptococcal meningitis has an atypical presentation in immunocompetent people, which poses a challenge in diagnosis. Furthermore, pet bird exposure is often overlooked as a source of cryptococcal infection. Higher suspicion of cryptococcal infection in the setting of nonspecific symptoms and known pet bird exposure may lead to an earlier diagnosis and, therefore, earlier treatment, potentially reducing neurological sequelae.
